# Adrenal Ganglioneuroblastoma in Adults: A Case Report and Review of the Literature

**DOI:** 10.1155/2017/5796236

**Published:** 2017-06-21

**Authors:** Stefano Benedini, Giorgia Grassi, Carmen Aresta, Antonietta Tufano, Luca Fabio Carmignani, Barbara Rubino, Livio Luzi, Sabrina Corbetta

**Affiliations:** ^1^Department of Biomedical Sciences for Health, Università degli Studi di Milano, Milan, Italy; ^2^Endocrinology Unit, IRCCS Policlinico San Donato, San Donato Milanese, Italy; ^3^Urology Department, IRCCS Policlinico San Donato, San Donato Milanese, Italy; ^4^Pathology Department, IRCCS Policlinico San Donato, San Donato Milanese, Italy; ^5^Endocrinology Service, Department of Biomedical Sciences for Health, University of Milan, IRCCS Istituto Ortopedico Galeazzi, Milan, Italy

## Abstract

Incidentally discovered adrenal masses are very common given the increased number of imaging studies performed in recent years. We here report a clinical case of a 20-year-old woman who presented with left flank pain. Ultrasound examination revealed a contralateral adrenal mass, which was confirmed at computed tomography (CT) scan. Hormonal hypersecretion was excluded. Given the size (11 × 10 × 7 cm) and the uncertain nature of the mass, it was surgically removed and sent for pathological analyses. Conclusive diagnosis was ganglioneuroblastoma. Ganglioneuroblastoma is an uncommon malignant tumor, extremely rare in adults, particularly in females. This neoplasm is frequently localized in adrenal gland.

## 1. Introduction

One of the most common unexpected findings revealed by imaging studies is an adrenal mass, called incidentaloma, which occurs in about 2–4% of the radiological studies performed for other reasons [1]. The preliminary evaluation of the mass is aimed to distinguish benign from malign lesions and to exclude hormonal hypersecretion. Most adrenal masses are small nonfunctioning adrenocortical adenomas, which do not require treatment or follow-up. Nevertheless, if the mass shows hormonal hypersecretion or malignancy is suspected, surgery is recommended [2].

Ganglioneuroblastoma (GNB) represents a rare cause of adrenal tumor in adults. Preoperative suspicion is challenging and the final diagnosis is often made by the pathologist after surgical removal.

## 2. Case Presentation

**Figure 1:**
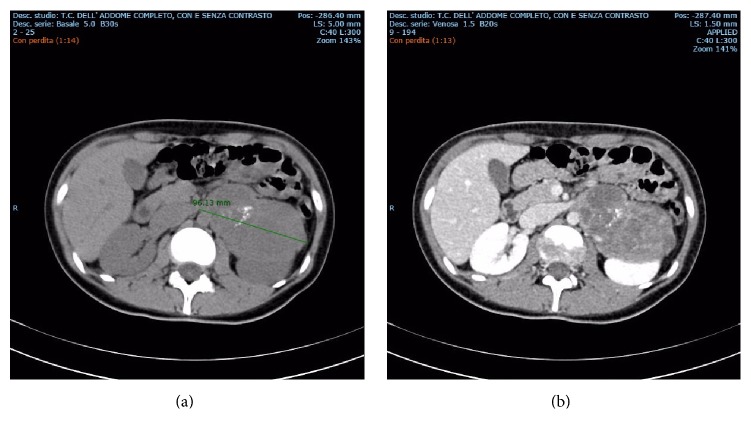
Abdominal CT scan with and without contrast enhancement: presence of a big and heterogeneous mass with calcification in left adrenal lodge (b). Dynamic analysis revealed a progressive and modest contrast enhancement in venous phase (a).

A 20-year-old Caucasian woman was admitted through the Emergency Department for right flank pain. Abdominal ultrasound examination was performed and a contralateral adrenal mass was incidentally found. Subsequently, the patient underwent computed tomography (CT) to confirm the finding. The mass in left adrenal lodge was solid and measured 11 × 10 × 7 cm, showing heterogeneous density (varying 17–40 HU) and calcifications (Figure 1). Dynamic analysis revealed a progressive and modest contrast enhancement in venous phase.

The patient was addressed to the Endocrine Unit for biochemical evaluation of the adrenal mass.

She was in good clinical conditions and the complete physical examination was negative; in particular, no Cushing stigmata or hirsutism was present. Her height was 153 cm, weight 48.5 kg (BMI 20.7 Kg/m^2^), blood pressure was 110/70 mmHg, pulse rate 64 beats/min, and SpO_2_ 99% (room air). There was no family history of relevant morbidities. She was active smoker and suffered from patent foramen ovale of the heart and focal nodular hyperplasia of the liver.

Results of the complete blood count, plasma levels of electrolytes, tests of coagulation, kidney, liver, and thyroid function were normal. Adrenal function evaluation revealed that urinary metanephrines and normetanephrines in the normal range, DHEA-S 1500 ng/ml (350–4300), aldosterone 457.2 pg/ml (37–150), renin 1.5 ng/ml/h (1.0–2.4), aldosterone-renin ratio 30.48, basal cortisol, and 17-OH-progesterone were normal both in basal condition and after stimulation with ACTH 250 mcg. Regrettably the patient was on contraceptive estroprogestinic therapy at the time of hormonal evaluation.

Considering the size and the undetermined radiological features, the adrenal mass met the criteria for surgical removal according the most recent international guidelines [2].

The patient was admitted to the Department of Urology where she underwent transabdominal adrenalectomy. The adrenal mass incorporated the renal hilum, aorta, and superior mesenteric arteria; therefore intraoperative decision to perform additional left nephrectomy was taken. There were no complications after surgery.

**Figure 2:**
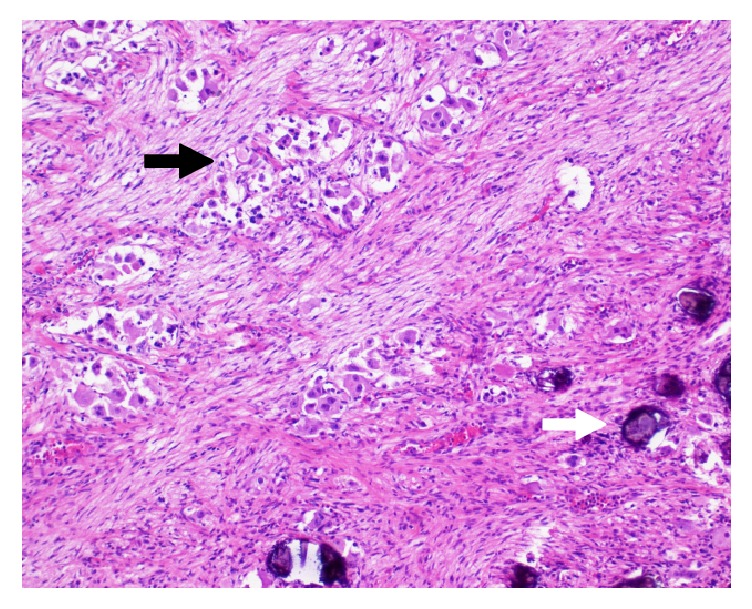
Histological examination (hematoxylin and eosin stain, 10x): high proportion of ganglion cells (black arrow) in spindle stroma and dystrophic calcification (white arrow).

The surgical sample sent for pathological examination included left kidney, left adrenal gland, and two lymph nodes (celiac and paraaortic). The tumor grossly was grey and multilobulated, replaced the entire adrenal gland, measured 11 × 10 × 7 cm, weighed 195 g, and incorporated arterial and venous vessels of renal hilum and sparing renal parenchyma. The histological report described a spindle cell stroma in a fibrillary matrix interspersed with scattered nests of primitive neuroblasts and high proportion of differentiating elements (ganglion cells) (Figure 2), placing the tumor in a favorable subgroup (ganglioneuroblastoma intermixed). Localization was found in both lymph nodes. These findings were consistent with intermixed stroma-rich ganglioneuroblastoma (GNB) according to Shimada et al., arising from the adrenal and with metastatic extension to ipsilateral lymph nodes [3]. The mitosis-karyorrhexis index (MKI) was <2%. In this case, any N-MYC amplification was detected and any deletion of the short arm of the chromosome 1. Chemotherapy was not proposed based on the favorable histology. Any disease recurrence occurred in the 21-month follow-up from surgery.

## 3. Discussion

Incidentally discovered adrenal masses are becoming more common with the imaging technological advances and the increased number of imaging studies performed. Nowadays the prevalence of adrenal incidentalomas in radiological studies has come close to the autoptic data: approximately 2–4% in adult age, increasing up to 10% in elderly population. Their differential diagnosis must consider a wide range of pathologies (Table 1). The prevalence of different etiologies varies among the studies; however, it is likely that the majority consists in nonfunctioning adenomas. Other frequently reported lesions are cortisol secreting adenomas, pheochromocytomas, primitive carcinomas, and distant metastatic lesions. In a study including 1111 adult patients with adrenal incidentalomas, GNB was diagnosed in only one case [4]. It is important to point out that the majority of adrenal lesions do not come to surgery; therefore pathological diagnoses of most adrenal incidentalomas remain unknown [1].

Peripheral neuroblastic tumors (PNTs) are a group of tumors arising from sympathetic ganglion cells. In two-thirds of the cases, PNTs arise in the adrenal gland or the retroperitoneal paravertebral ganglia. PNTs represent one of the most frequent solid tumors in children, while the occurrence in adults is very rare. Overall survival in infants is very high (91%) and progressively declines parallel to the increased age at diagnosis. In a study performed on RARECAREnet and involving very few cases, 5-year survival was reported to be 48% in adolescents (15–24 years) and 40% in adults (25–64 years) [5]. The vast majority of PNTs are sporadic and family history is reported only in a small percentage of cases [6]. Conditions such as Hirschsprung's disease and central hypoventilation, Turner syndrome, and Neurofibromatosis 1 seem to confer an increased risk of developing neuroblastic tumors, according to the literature [7–9]. No association with patent foramen ovale or focal nodular hyperplasia has been reported. PNTs are made up of two components: neuroblastic cells, with different degrees of differentiation, and Schwannian cells. The International Neuroblastoma Pathology Classification (INPC) distinguishes four pathological groups according to the different proportion of ganglion and Schwann cells: neuroblastoma (Schwannian stroma-poor, undifferentiated/poorly, differentiated/differentiating), ganglioneuroblastoma intermixed (Schwannian stroma-rich), ganglioneuroma (Schwannian stroma-dominant), and ganglioneuroblastoma nodular (composite Schwannian stroma-rich/stroma-dominant/stroma-poor) [3]. Clinic presentation is variable and the most common symptoms include pain or compression of the abdominal viscera. Metastatic dissemination occurs in about 40% of patients and involves more frequently bone and bone marrow. Catecholamines secretion is documented in more than 70% of cases [10]. The International Neuroblastoma Risk Group Consensus Pretreatment Classification Scheme defines prognosis and design treatment programs based on the stage of the tumor (according to the International Neuroblastoma Risk Group Staging System, INRGSS), age at diagnosis, pathology (INPC), and gene expression abnormalities (MYCN gene amplification, 11q aberration, and ploidy) (Table 2) [11]. Therapeutic modalities include surgery, radiotherapy, and chemotherapy combined on the basis of the individual patient. Very low risk group is treated only with surgery, followed by observation. Radiotherapy and chemotherapy are reserved for the higher risk groups, combined in different protocols [5].

Revision of the published literature in PubMed retrieved 15 cases of adult-onset adrenal GNB (Table 3) [12, 13]. The majority of patients were male and mean age at diagnosis was 38.9 years (21–67 years). Clinic was not specific and often represented by pain or other symptoms due to compression. Catecholamines secretion was documented only in 4 cases. Left adrenal gland was more frequently involved, and in one case bilateral tumors were reported. Imaging features of GNB varied from oval and homogeneous masses to heterogeneous, infiltrating, and calcified lesions. Because most of the lesions grew silently, at time of diagnosis big masses were found (mean size 10.44 cm). In locally advanced PNTs with potentially associated surgery-related complications, presurgical chemotherapy should be administered in order to shrink the tumor and enable safe resection saving other abdominal viscera involved [14]. Neuroblastic origin of the tumor was suspected preoperatively in just few cases, suggesting that though the radiological appearances of adrenal GNB have been described in detail [15], the preoperative diagnosis remains challenging and may be misleading. Consequently patients were addressed straight to surgery and the definitive etiology was histologically defined. Metastases were found at diagnosis in half of the patient and occurred in lymph nodes, liver, or bone marrow. The presence of metastases does not seem to correlate with the size or the histopathological subtype of the tumor. Most of the patients were treated only with surgery, showing no recurrence during follow-up (mean follow-up duration 20.9 months). Metastases were detected after 2.5 years in a patient who refused radio- and chemotherapy after surgical removal. One patient died 3 months after diagnosis due to heart failure.

No long-term data in adults have been reported due to the small series of patients.

There is no evidence about the most appropriate follow-up for adrenal GNB in adults.

## Figures and Tables

**Table 1:** Causes of adrenal masses.

*(i) Cystic masses*: endothelial cyst, pseudocyst, and hydatid cyst
*(ii) Solid masses*: adenoma, nodular hyperplasia, carcinoma, metastases, pheochromocytoma, neuroblastic tumors, neurofibroma, schwannoma, leiomyoma, angiosarcoma, hamartoma, tuberculoma, and amyloidosis
*(iii) Fat-containing masses*: lipoma and myelolipoma

Modified from Arnaldi and Boscaro [1].

**Table 2:** International Neuroblastoma Risk Group Consensus Pretreatment Classification Scheme.

INRG Stage	Age (months)	INPC group	Grade of differentiation	MYCN gene	11q aberration	Ploidy	Pretreatment risk group
*L1/L2*		GN maturing; GNB intermixed					Very low

*L1* Localized tumor confined to one body compartment and with absence of image-defined risk factors		Any, except GN maturing or GNB intermixed		NA Amplified			Very low High

*L2* Locoregional tumor with presence of one or more image-defined risk factor	<18	Any, except GN maturing or GNB intermixed		NA	No		Low
					Yes		Intermediate
	≥18	GNB nodular; NB	Differentiating	NA	No		Low
					Yes		Intermediate
			Poorly differentiated or undifferentiated	NA			
				Amplified			High

*M* Distant metastatic disease (except MS)	<18			NA		Hyperploid	Low
	<12			NA		Diploid	Intermediate
	12–18			NA		Diploid	Intermediate
	<18			Amplified			High
	≥18						High

*MS* Metastatic disease confined to skin, liver, and/or bone marrow in children <18 months				NA	no		Very low
					yes		High
				Amplified			High

INRG: International Neuroblastoma Risk Group; INCP: International Neuroblastoma Pathology Classification; GN = ganglioneuroma; GNB = ganglioneuroblastoma; NB = neuroblastoma; NA: not amplified; modified from Cohn et al. [11].

**Table 3:** Reported cases of adult-onset adrenal GNB in literature.

First Author (year)	Age (years)	Gender	Symptoms	Size (cm)	Side	Imaging	Hormonal activity	Metastases	Preliminary diagnosis	Histopathology	Treatment	Follow-up
Butz (1940)	25	M						Liver				
Cameron (1967)	58	F	Diarrhea		Right		↑ urinary catecholamines, vanilmandelic acid and homovanilmandelic acid	Absent	Pheochromocytoma		Surgery	3.5 years, no recurrence
Takahashi (1988)	21	M	Asymptomatic	8,8	Left		↑ urinary vanilmandelic acid	Lymph nodes	Neuroblastoma		Surgery + RT + CT	8 months, no recurrence
Koizumi (1992)	47	F	Fatigue, low back pain	9	Right	Heterogeneous	↑ urinary catecholamines, vanilmandelic acid and homovanilmandelic acid	Bone marrow	None		None	3 months, dead
Higuchi (1993)	29	M		11			↑ urinary catecholamines	Bone marrow			Surgery	10 months, no recurrence
Hiroshige (1995)	35	M	Asymptomatic	10	Left	Heterogeneous and calcifications	None	Absent	Carcinoma, neuroblastoma		Surgery	2 years, no recurrence
Mehta (1997)	22	M		9	Bilateral						Surgery	
Rousseau (1998)		F			Left			Liver			Surgery + RT + CT	
Fujiwara (2000)	25	M	Headache, palpitations, hypertension, weight loss	9	Left	Ovalar, heterogeneous, calcifications	None	Absent	Pheochromocytoma	GNB intermixed + pheochromocytoma	Surgery	5 years, no recurrence
Slapa (2002)	20	F	Asymptomatic	18			None	Absent			Surgery	1 year, no recurrence
Koike (2003)	50	M	Asymptomatic	4,5	Right	Ovalar, necrotic central area	None		Pheochromocytoma, adrenal malignancy, neuroblastic tumor		Surgery	2.5 years, no recurrence
Gunlusoy (2004)	59	M	Right flank and epigastric pain, malaise, anemia, weight loss, microscopic hematuria	12	Right	Lobulated, necrotic areas	None	Lymph nodes	None		Surgery	
Mizuno (2010)	53	M	Increased frequency of urination	11	Right	Smooth margins, homogeneous	None	Lumbar spine	None	GNB nodular	Surgery	Recurrence after 2.5 years
Bolzacchini (2015)	63	M	Asymptomatic	5	Left	Irregular margins, heterogeneous	None	Absent	None	GNB nodular	Surgery	6 months, no recurrence
Qiu (2015)	27	F	Pain	11	Left	Ovalar, cystic-solid	None	Absent	Pheochromocytoma	GNB intermixed	Surgery	5 months, no recurrence
Present case (2015)	21	F	Asymptomatic	11	Left	Lobulated, heterogeneous, calcifications	None	Lymph nodes	Adrenal carcinoma, leiomyosarcoma	GNB intermixed	Surgery	21 months, no recurrence

Modified from Bolzacchini et al. [12, 13].
